# Discordant identification of pediatric severe sepsis by research and clinical definitions in the SPROUT international point prevalence study

**DOI:** 10.1186/s13054-015-1055-x

**Published:** 2015-09-16

**Authors:** Scott L. Weiss, Julie C. Fitzgerald, Frank A. Maffei, Jason M. Kane, Antonio Rodriguez-Nunez, Deyin D. Hsing, Deborah Franzon, Sze Ying Kee, Jenny L. Bush, Jason A. Roy, Neal J. Thomas, Vinay M. Nadkarni, for the SPROUT Study Investigators and Pediatric Acute Lung Injury and Sepsis Investigators (PALISI) Network

**Affiliations:** Division of Critical Care Medicine, Department of Anesthesia and Critical Care, The Children’s Hospital of Philadelphia, University of Pennsylvania Perelman School of Medicine, Philadelphia, PA USA; Division of Pediatric Critical Care, Janet Weis Children’s Hospital at Geisinger Health System, Danville, PA USA; Department of Pediatrics, Section of Critical Care, University of Chicago Medicine, Comer Children’s Hospital, Chicago, IL USA; Division of Pediatric Emergency and Critical Care, Hospital Clínico Universitario de Santiago, Santiago de Compostela, Spain; Division of Pediatric Critical Care Medicine, New York Presbyterian Hospital, Weill Cornell Medical School, New York, NY USA; Division of Critical Care Medicine, Department of Pediatrics, Lucile Packard Children’s Hospital, Stanford University, Palo Alto, CA USA; Department of Pediatric Pulmonology, University Malaya Medical Centre, University of Malaya, Kuala Lumpur, Malaysia; Center for Clinical Epidemiology and Biostatistics, Department of Biostatistics and Epidemiology, University of Pennsylvania Perelman School of Medicine, Philadelphia, PA USA; Division of Pediatric Critical Care Medicine, Penn State Hershey Children’s Hospital, Penn State University College of Medicine, Hershey, PA USA

## Abstract

**Introduction:**

Consensus criteria for pediatric severe sepsis have standardized enrollment for research studies. However, the extent to which critically ill children identified by consensus criteria reflect physician diagnosis of severe sepsis, which underlies external validity for pediatric sepsis research, is not known. We sought to determine the agreement between physician diagnosis and consensus criteria to identify pediatric patients with severe sepsis across a network of international pediatric intensive care units (PICUs).

**Methods:**

We conducted a point prevalence study involving 128 PICUs in 26 countries across 6 continents. Over the course of 5 study days, 6925 PICU patients <18 years of age were screened, and 706 with severe sepsis defined either by physician diagnosis or on the basis of 2005 International Pediatric Sepsis Consensus Conference consensus criteria were enrolled. The primary endpoint was agreement of pediatric severe sepsis between physician diagnosis and consensus criteria as measured using Cohen’s κ. Secondary endpoints included characteristics and clinical outcomes for patients identified using physician diagnosis versus consensus criteria.

**Results:**

Of the 706 patients, 301 (42.6 %) met both definitions. The inter-rater agreement (κ ± SE) between physician diagnosis and consensus criteria was 0.57 ± 0.02. Of the 438 patients with a physician’s diagnosis of severe sepsis, only 69 % (301 of 438) would have been eligible to participate in a clinical trial of pediatric severe sepsis that enrolled patients based on consensus criteria. Patients with physician-diagnosed severe sepsis who did not meet consensus criteria were younger and had lower severity of illness and lower PICU mortality than those meeting consensus criteria or both definitions. After controlling for age, severity of illness, number of comorbid conditions, and treatment in developed versus resource-limited regions, patients identified with severe sepsis by physician diagnosis alone or by consensus criteria alone did not have PICU mortality significantly different from that of patients identified by both physician diagnosis and consensus criteria.

**Conclusions:**

Physician diagnosis of pediatric severe sepsis achieved only moderate agreement with consensus criteria, with physicians diagnosing severe sepsis more broadly. Consequently, the results of a research study based on consensus criteria may have limited generalizability to nearly one-third of PICU patients diagnosed with severe sepsis.

**Electronic supplementary material:**

The online version of this article (doi:10.1186/s13054-015-1055-x) contains supplementary material, which is available to authorized users.

## Introduction

Sepsis is a leading cause of death in children worldwide, responsible for an estimated 75,000 hospitalizations annually in the United States and nearly 50 % of all childhood hospital deaths worldwide [1–5]. Within the spectrum of this syndrome, *severe sepsis* refers to children with shock or other organ dysfunction and is the high-risk group targeted for interventional studies in the pediatric intensive care unit (PICU) [6].

Investigators in clinical trials of severe sepsis face important challenges that have contributed to high failure rates for many promising novel therapies, with few attempts to include children [7]. One fundamental issue is that the sepsis syndrome is characterized by non-specific physiologic abnormalities that encompass a heterogeneous population. Consensus criteria for pediatric sepsis were therefore established to facilitate consistent enrollment across research studies [6]. Many of these criteria have since been adopted for use in clinical practice [8]; however, published reports have demonstrated only moderate overlap of physician diagnosis of severe sepsis with consensus criteria [9, 10]. These findings raise concern that many children diagnosed and treated for severe sepsis in clinical practice may have important physiologic—and outcome—differences from those studied in interventional trials [9, 10].

The degree to which a study population is representative of patients diagnosed and treated in clinical practice has a major impact on the external validity of a study [11–13]. Although physician diagnosis serves clinical practice and consensus criteria were intended primarily for research, the alignment of these two methods to identify children with severe sepsis will significantly impact the extent to which study results translate into effective care at the bedside. Moreover, criteria that purposely define a disorder as a decompensated state at one extreme of the entire spectrum—as the existing pediatric consensus definitions currently do for severe sepsis and septic shock—may hinder early clinical diagnosis or delay enrollment in a clinical trial. Although the inherent challenge between research efficacy and clinical effectiveness is not limited to sepsis [14–17], understanding the extent to which consensus criteria for pediatric severe sepsis are in agreement with clinical practice will help to improve understanding of the utility of these criteria and to identify ways to improve both physician diagnosis and consensus definitions. To date, the agreement of physician diagnosis with consensus criteria for pediatric severe sepsis has not been evaluated in a large-scale setting.

The Sepsis PRevalence, OUtcomes, and Therapies (SPROUT) study researchers screened nearly 7000 PICU patients for severe sepsis at 128 sites across 26 countries using consensus criteria [18]. In addition, the attending physician caring for each patient provided an independent diagnostic assessment for severe sepsis. Using these data, we determined the level of agreement between consensus criteria [6] and attending physician diagnostic assessment (“physician diagnosis”) for pediatric severe sepsis. We hypothesized that agreement would be moderate at best, and thus we aimed to compare differences in patient characteristics, treatment strategies, and outcomes for children identified as having severe sepsis by consensus criteria versus physician diagnosis.

## Material and methods

### Study design

SPROUT was a prospective point prevalence study performed at 128 PICUs in 26 countries over the course of 5 study days spaced over 1 year [18]. Sites were recruited by open invitation through established research networks, and participation was voluntary. Ethical approval was obtained at all sites, and waiver of informed consent was granted at all but three sites, at which written consent was required for data collection (see Additional file 1 for a list of all approving ethical bodies). The details of the SPROUT study methodology have been published previously [18].

### Study population

All patients <18 years of age being treated in a participating PICU at 9:00 am local time on each study day were screened for severe sepsis using a standardized form incorporating the 2005 International Pediatric Sepsis Consensus Conference criteria: (1) at least two systemic inflammatory response syndrome criteria; (2) confirmed or suspected invasive infection; and (3) cardiovascular dysfunction, acute respiratory distress syndrome (ARDS), or at least two organ dysfunctions [6]. The subset of patients with septic shock defined by cardiovascular dysfunction were included within the spectrum of severe sepsis. Only data available within the 24 h preceding the 9:00 am study day time were considered for screening, yielding a study cohort with active severe sepsis. Patients who were ≥18 years of age, corrected gestational age <42 weeks, or had surgery involving cardiopulmonary bypass in the preceding 5 days were excluded.

To ascertain physician diagnosis of severe sepsis, an investigator at each site provided a list of patients, using a standardized form, to the attending physician of record on each study day. Attending physicians were instructed as follows: “In your clinical opinion, does this patient (yes or no) meet criteria for severe sepsis and/or septic shock when considering data only from the past 24 h (i.e., 9:00 am yesterday to 9:00 am today)?” Attending physicians who provided diagnoses were not involved with screening of patients for severe sepsis by consensus criteria. Similarly, site investigators screening for consensus criteria were blinded to physician diagnosis.

### Data collection

Data were collected about demographics, comorbid conditions, admission source, laboratory results, and therapies within a 48-h window around the study day (9:00 am before to 9:00 am after the study day). Definitions for the primary site of infection were adapted from published criteria [18]. The day of severe sepsis recognition was identified as the first calendar day on which a patient met consensus conference criteria for severe sepsis [6], and the presence of new or progressive multiorgan dysfunction syndrome (NPMODS) was measured for 7 days following severe sepsis recognition [19–21]. New MODS was defined as no or one organ dysfunction at sepsis recognition with subsequent development of at least two organ dysfunctions. Progressive MODS was defined as existing multiorgan dysfunction syndrome (MODS) (at least two organ dysfunctions) at sepsis recognition with development of at least one other concurrent organ dysfunction. For severe sepsis screening, organ dysfunctions were defined using pediatric sepsis consensus conference criteria [6], whereas more stringent criteria were used to define NPMODS [21]. For severity of illness, the Pediatric Index of Mortality (PIM)-3 score [22] was calculated at PICU admission, whereas the Pediatric Logistic Organ Dysfunction (PELOD) score [23] was calculated on the study day.

Patients with severe sepsis were followed for 90 days or until death or hospital discharge. Outcomes included vasoactive- and ventilator-free days from day of severe sepsis recognition through day 28, NPMODS, change in functional status from admission to hospital discharge (using the Pediatric Overall Performance Category [POPC] 1–6 ordinal scale [24]), and all-cause mortality at PICU discharge. Patients surviving to hospital discharge were classified as having “at least mild disability” for any increase in POPC and “at least moderate disability” if discharge POPC was 3 or higher and increased by at least 1 from baseline [25].

All data were recorded and managed using standardized case report forms within the web-based Research Electronic Data Capture (or REDCap) system, a secure database that provides an intuitive interface for validated data entry and audit trails for tracking data [26]. The methods used to ensure data quality and confidentiality have been published previously [18].

The primary outcome was the level of agreement in identification of pediatric severe sepsis between physician diagnosis and consensus criteria. Secondary outcomes included differences in patient characteristics, therapies used, and clinical outcomes between patients identified using physician diagnosis versus consensus criteria.

### Statistical analysis

Analyses were performed using STATA software (version 12.1; College Station, TX, USA). Data are presented as medians with interquartile ranges for continuous variables and frequencies with percentages for categorical variables. Comparisons between groups were performed using the Kruskal-Wallis test for continuous variables, and categorical variables were compared using Fisher’s exact test. For variables in which significant differences were identified across all three groups, pairwise comparisons were performed using Wilcoxon rank-sum or Fisher’s exact tests for continuous or categorical variables, respectively. The level of agreement between the two methods to identify patients with severe sepsis was quantified using percentage agreement and Cohen’s κ. Multivariable logistic regression was used to test the independent association of each definition of severe sepsis with PICU mortality after controlling for patient-level variables found to be significantly different across groups. Status as a developed (North America, Australia/New Zealand, and Europe) or resource-limited (Asia, Africa, and South America) region did not provide significant effect modification when tested as an interaction term with definition of severe sepsis (*p* = 0.36), but it was included as a confounder in the final logistic regression model. Statistical significance was defined as a *p* value <0.05.

## Results

**Fig. 1:**
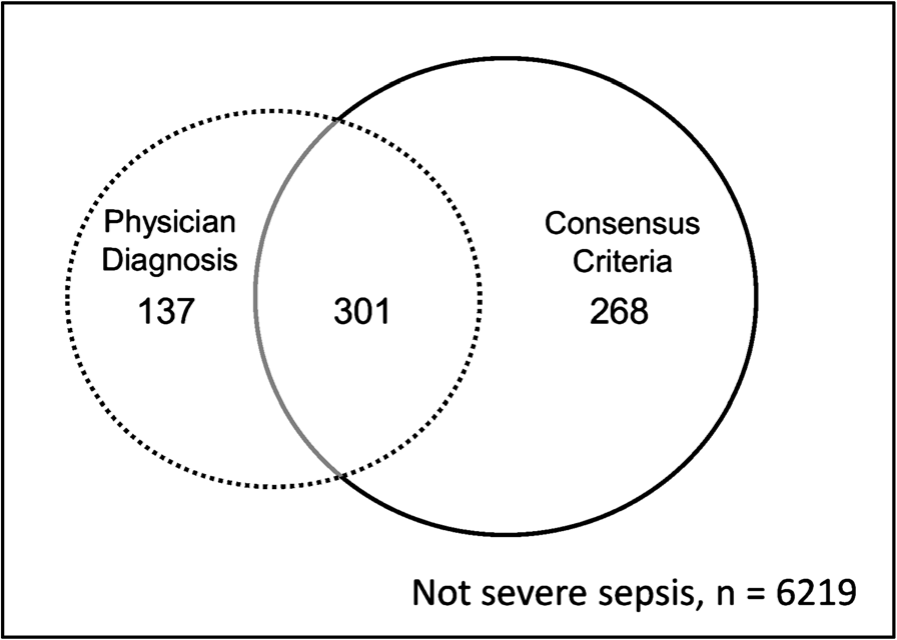
Venn diagram of the overlap between patients identified with severe sepsis by attending physician diagnostic assessment (“physician diagnosis”) and the 2005 International Pediatric Sepsis Consensus Conference (“consensus criteria”). Of the 6925 pediatric intensive care unit patients screened, 706 were identified with severe sepsis but only 301 (43 %) were concurrently identified by both physician diagnosis and consensus criteria (κ 0.57 ± 0.02)

Over the 5 study days, 6925 PICU patients were screened across all sites. In total, 706 were identified with severe sepsis by either consensus criteria or physician diagnosis, with 137 patients (19.4 %) identified by physician diagnosis only, 301 (42.6 %) by both definitions, and 268 (38.0 %) by consensus criteria only (Fig. 1). The percentage agreement between consensus criteria and physician diagnosis for identifying severe sepsis in the entire cohort of screened patients was 94 %, but it was only 43 % for identifying severe sepsis among the 706 patients with sepsis by either definition. The inter-rater agreement between physician diagnosis and consensus criteria achieved a moderate κ ± standard error of 0.57 ± 0.02. The percentage agreement between physician diagnosis and consensus criteria varied significantly across geographic regions (*p* < 0.001). Agreement was lowest in North America (31 % agreement of 444 patients identified with severe sepsis by any criteria); moderate in Australia and New Zealand (45 %) and Europe (51 %); and highest in Asia (72 %), Africa (72 %), and South America (85 %). The percentage agreement was 44 %, 49 %, 45 %, 38 %, and 39 % across study days 1–5, respectively, suggesting that clinicians did not alter their diagnostic assessment to better comply with consensus criteria over time.

Of the 438 total patients with a physician diagnosis of severe sepsis, 31 % were not concurrently identified with severe sepsis by consensus criteria and thus would have been ineligible to participate in a clinical trial that enrolled patients based on the criteria established by consensus guidelines. Moreover, 47 % of the patients identified as having severe sepsis by consensus criteria—and thus potentially eligible for a clinical trial—were not simultaneously considered to have severe sepsis by physician diagnosis.

Characteristics of 704 of the patients identified with severe sepsis by physician diagnosis alone, by both definitions, or by consensus criteria alone are shown in Table 1. Data could not be obtained for two patients (both identified by consensus criteria alone) who did not consent to data collection at one site that required informed consent for this purpose. Patients identified only by physician diagnosis were younger (*p* = 0.008), had a lower severity of illness (lower PIM-3 [*p* < 0.001] and PELOD [*p* < 0.001] scores), and a lower proportion of cardiovascular (*p* = 0.006) and hematologic dysfunction (*p* < 0.001) at sepsis recognition than patients identified by consensus criteria alone or both definitions. Patients identified by consensus criteria alone were less likely to be previously healthy (*p* = 0.002) and more likely to have neuromuscular comorbidities (*p* < 0.001) than patients identified by physician diagnosis alone or both definitions. Patients identified by consensus criteria alone were more likely to have respiratory (*p* = 0.004) or unknown primary site of infection (*p* = 0.002) and less likely to have primary bloodstream infection (*p* < 0.001).

**Table 1:** Characteristics of patients identified with severe sepsis by physician diagnosis, consensus criteria, or both

Characteristic	Physician diagnosis	Both criteria	Consensus criteria	*p* value*
N	137	301	266	
Age (yr)	1 (0.5–7)	3 (0.7–10)	3 (0.7–11)	0.03
Sex, n (%)				0.74
Male	76 (55)	164 (55)	138 (52)	
Female	61 (45)	137 (45)	128 (48)	
Comorbid conditions, n (%)				
Respiratory	54 (39)	73 (24)	99 (37)	0.001
Gastrointestinal	40 (29)	62 (21)	79 (30)	0.03
Cardiovascular	34 (25)	62 (21)	74 (28)	0.13
Genetic	35 (26)	54 (18)	61 (23)	0.14
Hematologic/immunologic	28 (20)	69 (23)	45 (17)	0.21
Neuromuscular	11 (8)	37 (12)	60 (23)	<0.001
Neoplastic	18 (13)	51 (17)	29 (11)	0.11
Prematurity	37 (12)	24 (18)	39 (15)	0.35
Metabolic	17 (12)	28 (9)	34 (13)	0.38
Renal	15 (11)	24 (8)	31 (12)	0.31
Solid organ/stem cell transplant	8 (6)	28 (9)	26 (10)	0.39
Comorbid conditions, n (%)				<0.001
None	30 (22)	85 (28)	43 (16)	
1	21 (15)	77 (26)	64 (24)	
≥ 2	86 (63)	21 (15)	159 (60)	
Admission POPC, n (%)				0.13
Good performance	70 (51)	170 (56)	120 (45)	
Mild disability	26 (19)	44 (15)	41 (15)	
Moderate disability	22 (16)	44 (15)	46 (17)	
Severe disability or coma	19 (14)	43 (14)	59 (22)	
PIM-3 score^a^	3.6 (1.4–5.5)	4.4 (1.9–9.2)	4.3 (1.8–9.9)	<0.001
PELOD score^b^	10 (1–11)	11 (2–13)	11 (2–12)	<0.001
Source of admission, n (%)				0.86
Emergency department^c^	41 (30)	89 (30)	78 (29)	
Hospital floor	39 (29)	92 (31)	66 (25)	
Operating room	12 (9)	28 (9)	22 (8)	
Other hospital^d^	39 (28)	77 (26)	89 (33)	
Organ dysfunction at sepsis recognition^e^				
Cardiovascular	51 (37)	182 (61)	106 (40)	<0.001
Respiratory	88 (64)	211 (70)	197 (74)	0.12
Renal	5 (4)	19 (6)	7 (3)	0.10
Hepatic	2 (1)	16 (5)	6 (2)	0.07
Hematologic	4 (3)	49 (16)	24 (9)	<0.001
Neurologic	9 (7)	28 (9)	33 (12)	0.17
Primary site of infection, n (%)				
Respiratory	60 (44)	101 (34)	127 (48)	0.002
Primary bloodstream	25 (18)	82 (27)	26 (10)	<0.001
Abdominal	15 (11)	31 (10)	16 (6)	0.11
Central nervous system	3 (2)	9 (3)	16 (6)	0.11
Genitourinary	7 (5)	12 (4)	9 (3)	0.67
Skin	4 (3)	16 (5)	4 (2)	0.04
Other	6 (4)	16 (5)	13 (5)	0.95
Unknown	17 (12)	34 (11)	55 (21)	0.006
Microbiology,^f,g^ n (%)				
Gram-positive bacteria	39 (28)	90 (30)	60 (23)	0.13
Gram-negative bacteria	27 (20)	95 (32)	63 (24)	0.02
Fungus	9 (7)	39 (13)	37 (14)	0.07
Virus	35 (26)	50 (17)	69 (26)	0.01
No organism identified	50 (36)	95 (32)	101 (38)	0.25

Data are presented as median (IQR) unless otherwise noted

*POPC* Pediatric Overall Performance Category, *ScvO*_*2*_ central venous oxygen saturation, *PIM-3* Pediatric Index of Mortality-3, *PELOD* Pediatric Logistic Organ Dysfunction, *PICU* pediatric intensive care unit

*Statistical comparisons using Kruskal-Wallis or Fisher’s exact test

^a^PIM-3 measured at time of PICU admission

^b^PELOD score calculated from data within a 48-h time window around the study day (9:00 am before to 9:00 am after the study day)

^c^Emergency department at the same hospital as the PICU

^d^Other hospital includes emergency department at another hospital

^e^Based on organ dysfunction criteria defined by the International Pediatric Sepsis Consensus Conference [6]

^f^Categories do not add up to 100 %, as some infections were polymicrobial

^g^Sources of positive isolates included blood, urine, cerebrospinal fluid, respiratory (nasopharynx, tracheal, bronchoalveolar lavage), stool, wound, and other normally sterile body fluids (pleural, pericardial, peritoneal)

Patients identified concurrently by both physician diagnosis and consensus criteria had lower platelet counts and higher blood lactate, C-reactive protein, and procalcitonin levels than patients identified by either criterion alone (all *p* < 0.01) (Table 2). Similarly, patients identified by both physician diagnosis and consensus criteria were also more likely to be treated with vasoactive infusions, albumin, blood products, granulocyte/granulocyte macrophage colony-stimulating factor, intravenous immunoglobulin, and renal replacement therapy than patients identified by either criterion alone (all *p* < 0.05) (Table 3).

**Table 2:** Laboratory values within the 48-h data collection window by definition of severe sepsis

Laboratory examination	Physician diagnosis		Both criteria		Consensus criteria		*p* value*
	Percent^a^	Median (IQR)	Percent^a^	Median (IQR)	Percent^a^	Median (IQR)	
WBC, max (10^3^/μl)	92 %	10.1 (7.3–16.6)	97 %	14.9 (7.5–22.7)	91 %	14.6 (8.8–20.8)	<0.001
Platelets, min (10^3^/μl)	91 %	148 (70–262)	96 %	95 (39–195)	89 %	157 (77–287)	<0.001
BUN, max (mg/dl)	83 %	13 (8–22)	93 %	21 (12–41)	92 %	18 (10–31)	<0.001
Creatinine, max (mg/dl)	92 %	0.4 (0.3–0.6)	97 %	0.5 (0.3–0.9)	97 %	0.4 (0.3–0.7)	<0.001
ALT, max (IU/L)	55 %	39 (25–67)	77 %	53 (28–123)	62 %	50 (26–110)	0.09
Total bilirubin, max (mg/dl)	53 %	0.7 (0.4–1.7)	73 %	1.0 (0.5–2.9)	59 %	0.7 (0.4–3.0)	0.02
Albumin, min (g/dl)	66 %	2.6 (2.2–3.0)	81 %	2.5 (2.2–3.0)	71 %	2.8 (2.3–3.3)	<0.001
INR, max	37 %	1.3 (1.2–1.7)	64 %	1.3 (1.2–1.8)	42 %	1.3 (1.1–1.8)	0.80
Lactate, max (mmol/L)	62 %	1.4 (1.1–2.0)	75 %	2.0 (1.2–3.8)	62 %	1.6 (1.1–2.8)	0.001
ScvO_2_, min (%)	28 %	70 (57–75)	46 %	65 (52–74)	30 %	65 (49–73)	0.50
PaO_2_/FiO_2_ ratio, min (mmHg)	47 %	138 (89–233)	72 %	160 (100–236)	51 %	144 (87–275)	0.79
C-reactive peptide, max (mg/dl)	39 %	7.1 (2.1–17.8)	64 %	9.3 (2.8–20.8)	43 %	3.8 (1.6–9.7)	<0.001
Procalcitonin, max (ng/ml)	12 %	3.5 (1.1–11.6)	22 %	6.4 (1.4–23.0)	6 %	1.3 (0.24–5.4)	0.01

*max* maximum value within the 48-h window around the study day (9:00 am before to 9:00 am after the study day), *min* minimum value within the 48-h window around the study day (9:00 am before to 9:00 am after the study day), *PaO*_*2*_ */FiO*_*2*_ ratio of partial pressure of oxygen to fraction of inspired oxygen, *WBC* white blood cell count, *BUN* blood urea nitrogen, *ALT* alanine aminotransferase, *INR* international normalized ratio, *ScvO*_*2*_ central venous oxygen saturation

*Statistical comparisons using Kruskal-Wallis test

^a^Proportion of each group with available laboratory results

**Table 3:** Therapies used within the 48-h data collection window by definition of severe sepsis

Therapy	Physician diagnosis	Both criteria	Consensus criteria	*p* value*
Vasoactive infusions^a^	53 (39)	209 (69)	105 (39)	<0.001
Invasive mechanical ventilation	92 (67)	229 (76)	192 (72)	0.14
Corticosteroids	46 (36)	132 (44)	110 (41)	0.13
Albumin	24 (18)	90 (30)	45 (17)	<0.001
Blood products^b^	39 (28)	159 (53)	73 (27)	<0.001
Insulin^c^	4 (3)	32 (11)	25 (9)	0.02
G/GM-CSF	2 (1)	17 (6)	6 (2)	0.04
IVIG	7 (5)	28 (9)	10 (4)	0.02
RRT^d^	13 (9)	57 (19)	24 (9)	0.001
Plasma exchange	1 (1)	3 (1)	2 (1)	0.94
ECMO	3 (2)	14 (5)	16 (6)	0.23

Data presented as n (%)

*G/GM-CSF* granulocyte/granulocyte-monocyte colony stimulating factor, *IVIG* intravenous immunoglobulin, *RRT* renal replacement therapy, *ECMO* extracorporeal membrane oxygenation

*Statistical comparisons using Fisher’s exact test

^a^Includes dopamine >5 μg/kg/min, dobutamine >5 μg/kg/min, or any dose of epinephrine, norepinephrine, vasopressin, phenylephrine, milrinone, levosimendan, or vasodilator

^b^Includes packed red blood cells, platelets, fresh frozen plasma, cryoprecipitate, granulocytes, and whole blood

^c^Includes intravenous insulin by continuous infusion only

^d^Includes hemodialysis, all continuous renal replacement modalities, and peritoneal dialysis

PICU mortality was significantly lower for patients with only a physician diagnosis of severe sepsis (18 %) than with both definitions (27 %; *p* = 0.02) and trended lower than for consensus criteria alone (21 %; *p* = 0.29; Table 4). The proportion with NPMODS, at least mild disability, and at least moderate disability all trended lower for patients identified with severe sepsis by physician diagnosis alone, but none reached statistical significance. After controlling for age, PIM-3 score, number of comorbid conditions, and treatment in developed versus resource-limited regions, patients identified with severe sepsis by physician diagnosis only or by consensus criteria only did not have PICU mortality different from that of patients identified by both physician diagnosis and consensus criteria (Table 5).

**Table 4:** Clinical outcomes by definition of severe sepsis

Outcome	Physician diagnosis	Both criteria	Consensus criteria	*p* value*
Vasoactive-free days, median (IQR)	27 (22–28)	22 (6–26)	26 (17–28)	<0.001
Ventilator-free days, median (IQR)	19 (2–27)	16 (0–24)	18 (0–26)	0.07
NPMODS^a^	47 (34)	131 (44)	97 (36)	0.10
PICU mortality	23 (17)	82 (27)	57 (21)	0.042
At least mild disability^b^	24 (22)	67 (31)	50 (24)	0.15
At least moderate disability^c^	14 (13)	36 (17)	37 (18)	0.46

Data presented as n (%) unless otherwise noted

*IQR* interquartile range, *NPMODS* new or progressive multiorgan dysfunction syndrome, *PICU* pediatric intensive care unit

*Statistical comparisons using Kruskal-Wallis or Fisher’s exact test

^a^NPMODS considered starting the day after sepsis recognition

^b^Any increase in Pediatric Overall Performance Category from baseline to hospital discharge in the 534 hospital survivors

^c^Discharge Pediatric Overall Performance Category ≥3 and an increase of at least 1 from baseline in the 534 hospital survivors

**Table 5:** Multivariable logistic regression model of the association of severe sepsis definition with PICU mortality

Variable	Adjusted OR^a^	95 % confidence interval	*p* value
Severe sepsis definition			
Both criteria	Reference		
Physician diagnosis, only	0.64	0.37–1.11	0.12
Consensus criteria, only	0.73	0.48–1.12	0.15
Age, yr	0.99	0.95–1.02	0.45
PIM-3 score	1.04	1.03–1.06	<0.001
Comorbid conditions			
None	Reference		
1 comorbid condition	1.11	0.62–2.01	0.72
≥2 comorbid conditions	1.78	1.07–2.99	0.03
Developed region^b^	0.63	0.37–1.06	0.08

*OR* odds ratio, *PIM-3* Pediatric Index of Mortality-3

^a^Odds ratios are adjusted for all of the variables listed

^b^Sites included as “developed regions” were located in North America, Australia/New Zealand, and Europe

## Discussion

Across this large international network of PICUs, physician diagnosis and consensus criteria achieved only a moderate level of agreement in identifying critically ill children with severe sepsis. These findings suggest that the results of a research study with enrollment based only on current consensus criteria may not be generalizable to nearly one-third of pediatric patients diagnosed with severe sepsis in a PICU. Moreover, for nearly half of the patients identified with severe sepsis by consensus criteria, and thus eligible for clinical trials, a diagnosis of severe sepsis was not corroborated by the treating attending physician.

The data in this study demonstrate that PICU physicians worldwide commonly diagnose critically ill children with severe sepsis who do not meet consensus criteria. This finding is consistent with a prior report of a single-center study in the United States [10]. In general, patients with physician-diagnosed severe sepsis tended to be younger, less severely ill, and less likely to receive several sepsis-related therapies than patients who also met consensus criteria. These observations likely reflect the intention of the International Pediatric Sepsis Consensus Conference to identify a more severely ill subgroup of critically ill children with severe sepsis for enrollment in clinical trials [6]. It may therefore be appropriate for physicians to diagnose severe sepsis more often than consensus criteria, particularly in children with a lower severity of illness who may need fewer adjunctive sepsis-related therapies. Physicians may also have been recognizing and treating children with sepsis earlier in their illness course, at a point when they may not yet have manifested the physiologic and laboratory derangements required by consensus criteria. The loss of a statistical difference in PICU mortality between patients identified by physician diagnosis alone and those identified by both definitions after correcting for severity of illness is consistent with these suppositions. A lower threshold for diagnosing severe sepsis in clinical practice likely accounts for the generally better outcome metrics identified in epidemiologic studies using administrative databases [1, 2, 5] than has been reported in clinical trials [20, 27-29]. Our study therefore suggests caution when extrapolating results from epidemiologic, observational, and interventional studies to this less severely ill subgroup of patients who are diagnosed on the basis of physician impression and do not meet consensus criteria. Conversely, the absence of certain features, such as two or more systemic inflammatory response syndrome criteria [30, 31], hypotension only after 40 ml/kg of intravenous fluid, and laboratory derangements beyond arbitrary cutpoints (e.g., international normalized ratio >2), should not preclude the clinical diagnosis of severe sepsis and/or septic shock. A refined consensus definition that more closely matches the clinical definition and establishes a spectrum of risk concordant with outcome, similar to that of the recent at-risk, mild, moderate, and severe pediatric ARDS definitions [32], would benefit both research scientists and clinicians.

By quantifying the extent to which physician diagnosis and consensus criteria overlap, one can better understand the potential loss of efficacy as research findings are adopted at the bedside. Previous studies show that efficacious therapies in clinical trials are often extended to a broader patient population, with a more variable and perhaps even a distinct pathophysiology [14–17]. To our knowledge, this is the first multicenter, international study of the generalizability of consensus criteria for pediatric severe sepsis. Because only 69 % of patients with a physician’s diagnosis of severe sepsis met consensus criteria, it may not be appropriate to apply the results of a clinical trial to up to one-third of children treated for severe sepsis in a general PICU practice. Although a potential decrement from efficacy to effectiveness may not dissuade widespread implementation of a new therapy, enthusiasm could be altered if concerns over adverse effects, resource utilization, or costs were taken into consideration.

It is also noteworthy that nearly half of patients in the SPROUT study were identified as having severe sepsis by consensus criteria without corroboration by physician diagnosis. This likely reflects that both physician diagnosis and consensus criteria rely on relatively non-specific changes in physiology that are commonly found in critically ill children in general, not just in children with sepsis. Still, this observation highlights an opportunity to refine future iterations of consensus criteria and address variability in physician diagnosis to identify sepsis. Patients with preexisting neuromuscular disorders, such as cerebral palsy and epilepsy, were particularly likely to meet consensus criteria for severe sepsis despite an alternative clinical diagnosis. Many of these patients have dysautonomia or altered physiologic responses to illness that make it challenging to apply predefined rigid criteria. The higher rate of unknown primary site of infection suggests that consensus criteria may be less specific than physician diagnosis in identifying patients with a true infection. It is also possible that consensus criteria include PICU patients with a high severity of illness, namely those with shock and multiorgan system dysfunction caused by non-septic insults, who are commonly treated empirically for infection even if clinical suspicion is low. Alternatively, if physician diagnosis does not corroborate consensus criteria despite true presence of severe sepsis, there may be a higher rate of parental or clinician refusal to participate in research because the clinicians managing the patient may not agree on the diagnosis of concern. The incorporation of new biomarkers or novel microbiologic detection systems that improve early sensitivity and specificity for invasive infections into future revisions of the consensus criteria may improve alignment with physician diagnosis.

There are several limitations to this study. First, physician diagnosis and the application of consensus criteria to identify patients with severe sepsis could not be independently verified. The reasons why patients were not identified as having severe sepsis by one or both definitions were also not available. Although these concerns may have led to misclassification of some patients, it is unlikely that such a bias would have preferentially affected one group. Moreover, because we included only physicians practicing in a PICU setting, 89 % of whom were trained in pediatric critical care medicine, the level of agreement for other providers who commonly treat sepsis (e.g., emergency physicians) may differ from our results. A second limitation is that criteria used for physician diagnosis were not standardized. Although this may have increased variability in physician diagnosis across providers and sites, such an approach reflects that physicians often differ in their diagnostic approach. Third, the higher proportion of patients with available laboratory measures in the consensus criteria only and both definition groups may itself have increased the likelihood of identification by consensus criteria because, in the absence of available data, these measures were assumed to be within normal limits. Although it is more likely that sicker patients had directed laboratory testing performed than unsuspected organ dysfunction was discovered through routine laboratory testing, differential availability of laboratory results may have been a source of misclassification bias. Finally, because a “gold standard” does not exist, it is not possible to determine the degree to which either definition correctly identified pediatric patients with severe sepsis or compared the sensitivity and specificity of the two definitions.

## Conclusions

Physician diagnosis of pediatric severe sepsis achieved only a moderate level of agreement with consensus criteria across this multicenter, international point prevalence study. The results of a research study based on current consensus criteria may not be generalizable to nearly one-third of pediatric patients diagnosed with severe sepsis in a PICU. Further research is needed to understand the extent to which the specificity of consensus criteria and variability in physician diagnosis may be contributing to this discordant identification of pediatric severe sepsis. Attempts to better align consensus definitions with clinical diagnosis by establishing a continuous spectrum of illness severity are needed.

## Key messages

- Although consensus criteria for pediatric sepsis were established to facilitate consistent enrollment across research studies, the extent to which these criteria reflect physician diagnosis of severe sepsis, which underlies external validity for pediatric sepsis research, is not known.
- Of 6925 PICU patients screened at 128 PICUs in 26 countries, 706 patients were identified by physician diagnosis and/or consensus criteria as having severe sepsis.
- Only 301 patients (42.6 %) were identified by both physician diagnosis and consensus criteria (κ 0.57 ± 0.02).
- The 31 % of patients with physician-diagnosed severe sepsis who did not meet consensus criteria were younger, had a lower severity of illness, and a lower PICU mortality than those who met consensus criteria or both definitions.
- The results of a research study based on consensus criteria may have limited generalizability to nearly one-third of PICU patients diagnosed with severe sepsis.

## Additional file

Additional file 1:
**Approving ethical bodies at each study site.** (DOCX 16 kb)

## Abbreviations

ALT: alanine aminotransferase
ARDS: acute respiratory distress syndrome
BUN: blood urea nitrogen
ECMO: extracorporeal membrane oxygenation
G/GM-CSF: granulocyte/granulocyte macrophage colony-stimulating factor
INR: international normalized ratio
IQR: interquartile range
IVIG: intravenous immunoglobulin
MODS: multiorgan dysfunction syndrome
NPMODS: new or progressive multiorgan dysfunction syndrome
OR: odds ratio
PaO_2_/FiO_2_: ratio of partial pressure of oxygen to fraction of inspired oxygen
PELOD: Pediatric Logistic Organ Dysfunction score
PICU: pediatric intensive care unit
PIM: Pediatric Index of Mortality score
POPC: Pediatric Overall Performance Category
REDCap: Research Electronic Data Capture
RRT: renal replacement therapy
ScvO_2_: central venous oxygen saturation
SPROUT: Sepsis PRevalence, OUtcomes, and Therapies study
WBC: white blood cells
