# Supplementary material for: Discordant identification of pediatric severe sepsis by research and clinical definitions in the SPROUT international point prevalence study
Source: Crit Care. 2015 Sep 16;19(1):325. doi: 10.1186/s13054-015-1055-x (PMC4572676; doi:10.1186/s13054-015-1055-x)
Supplement: Additional file 1: — Approving ethical bodies at each study site. (DOCX 16 kb) [file 13054_2015_1055_MOESM1_ESM.docx]

Additional File 1: Approving ethical bodies at each study site

| **Site** | **Approving Ethical Committee** |
| --- | --- |
| **North America** |  |
| Montreal Children's Hospital-McGill | Montreal Children's Hospital-McGill |
| Sainte Justine Hospital | Sainte Justine Hospital |
| BC Children's Hospital | BC Children's Hospital |
| Hospital Cardiovascular de Puerto Rico y el Caribe | Hospital Cardiovascular de Puerto Rico y el Caribe |
| San Jorge Children’s Hospital | San Jorge Children’s Hospital |
| University Pediatric Hospital | University Pediatric Hospital |
| Akron Children's Hospital | The Children’s Hospital of Philadelphia^1^ |
| Amplatz Children’s Hospital | Amplatz Children’s Hospital |
| Arkansas Children’s Hospital | Arkansas Children’s Hospital |
| Boston Children’s Hospital | Boston Children’s Hospital |
| Children’s Hospital of Pittsburgh | Children’s Hospital of Pittsburgh |
| Children’s Hospital-Hackensack | Children’s Hospital-Hackensack |
| Children’s Mercy Hospital | Children’s Mercy Hospital |
| Children's Healthcare of Atlanta | Children's Healthcare of Atlanta |
| Children’s Hospital in Minnesota | Children’s Hospital in Minnesota |
| Children’s Hospital St. Paul | Children’s Hospital St. Paul |
| Children's Hospital Colorado | Children's Hospital Colorado |
| Children's Hospital New Orleans | Children's Hospital New Orleans |
| Children’s Hospital of Los Angeles | Children’s Hospital of Los Angeles |
| Children’s Hospital of Monmouth | Children’s Hospital of Monmouth |
| Children's Hospital of Wisconsin | Children's Hospital of Wisconsin |
| Children's National Medical Center | Children's National Medical Center |
| Cincinnati Children‘s Medical Center | The Children’s Hospital of Philadelphia^1^ |
| Connecticut Children's Medical Center | Connecticut Children's Medical Center |
| Dartmouth Hitchcock | The Children’s Hospital of Philadelphia^1^ |
| Dayton Children's Hospital | Dayton Children's Hospital |
| Dell Children's Medical Center | Dell Children's Medical Center |
| Diamond Children's Hospital | The Children’s Hospital of Philadelphia^1^ |
| Doernbecher Children's Hospital | Doernbecher Children's Hospital |
| Duke Children's Hospital | Duke Children's Hospital |
| El Paso Children's Hospital | El Paso Children's Hospital |
| Golisano Children’s Hospital | Golisano Children’s Hospital |
| Janet Weis/Geisinger | The Children’s Hospital of Philadelphia^1^ |
| Johns Hopkins | Johns Hopkins |
| Le Bonheur Children’s Hospital | Le Bonheur Children’s Hospital |
| Lucile Packard Children’s Hospital | Lucile Packard Children’s Hospital |
| Lurie Children's Hospital of Chicago | Lurie Children's Hospital of Chicago |
| Medical City Children's-Dallas | Medical City Children's-Dallas |
| Medical University of South Carolina | Medical University of South Carolina |
| Miami Children’s Hospital | Miami Children’s Hospital |
| Nationwide Children's Hospital | Nationwide Children's Hospital |
| Nemours/ Alfred I. duPont Children’s Hospital | The Children’s Hospital of Philadelphia^1^ |
| Penn State Hershey Medical Center | The Children’s Hospital of Philadelphia^1^ |
| Primary Children's Medical Center | Primary Children's Medical Center |
| Riley Hospital for Children | The Children’s Hospital of Philadelphia^1^ |
| Rush Children's Hospital | Rush Children's Hospital |
| Seattle Children's Hospital | Seattle Children's Hospital |
| St. Louis Children's Hospital | St. Louis Children's Hospital |
| Stony Brook University | Stony Brook University |
| Texas Children's Hospital | Texas Children's Hospital |
| The Children’s Hospital of Philadelphia | The Children’s Hospital of Philadelphia |
| Oklahoma University Medical Center | Oklahoma University Medical Center |
| University of California San Francisco | University of California San Francisco |
| University of Iowa | University of Iowa |
| Weill Cornell Medical Center | Weill Cornell Medical Center |
| Wolfson Children’s Hospital | Wolfson Children’s Hospital |
| Women and Children’s Hospital of Buffalo | Women and Children’s Hospital of Buffalo |
| Yale Children’s Hospital | Yale Children’s Hospital |
|  |  |
| **South America** |  |
| Hospital Durand | Hospital Durand |
| Clínica Infantil Colsubsidio | Clínica Infantil Colsubsidio |
| Hospital de San Jose | Hospital de San Jose |
| Hospital General de Medellín | Hospital General de Medellín |
| Hospital Military Central | Hospital Military Central |
| Pablo Tobón Uribe | Pablo Tobón Uribe |
| Clínica Las Condes | Clínica Las Condes |
| Clínica Santa María | Clínica Santa María |
| Hospital Luis Calvo Mackenna | Hospital Luis Calvo Mackenna |
| Hospital Padre Hurtado | Hospital Padre Hurtado |
|  |  |
| **Europe** |  |
| St. Luc University Hospital | St. Luc University Hospital |
| Masaryk University | Masaryk University |
| Klinikum Augsburg | Klinikum Augsburg |
| Aghia Sophia Children’s Hospital | Aghia Sophia Children’s Hospital |
| P. & A. Kyriakou Children’s Hospital | P. & A. Kyriakou Children’s Hospital |
| Bambino Gesu Area Rossa | Bambino Gesu Area Rossa |
| Bambino Gesu Children’s Hospital | Bambino Gesu Children’s Hospital |
| Bambino Gesu Pediatrico | Bambino Gesu Pediatrico |
| Lithuanian University of Health Sciences | Lithuanian University of Health Sciences |
| Radboud University Medical Centre | Radboud University Medical Centre |
| Polish Mother Memorial Hospital | Polish Mother Memorial Hospital |
| Szyszko Śląskiego University | Szyszko Śląskiego University |
| Hospital Prof Dr. Fernando Fonseca | Hospital Prof Dr. Fernando Fonseca |
| Centrol Hospitalar Lisboa Norte | Centrol Hospitalar Lisboa Norte |
| Children´s Hospital Miguel Servet | Children´s Hospital Miguel Servet |
| Hospital General Universitario Gregorio Marañón | Hospital General Universitario Gregorio Marañón |
| Hospital 12 de Octubre | Hospital 12 de Octubre |
| Hospital Clínico Universitario | Hospital Clínico Universitario |
| Hospital de la Sant Creu Sant Pau | Hospital de la Sant Creu Sant Pau |
| Hospital Universitario Madrid | Hospital Universitario Madrid |
| Hospital Carlos Haya Materno Infantil | Hospital Carlos Haya Materno Infantil |
| Hospital Sant Joan de Déu | Hospital Sant Joan de Déu |
| Hospital Universitario Donostia | Hospital Universitario Donostia |
| Hospital Universitario Salamanca | Hospital Universitario Salamanca |
| Hospital Virgen de la Arrixaca | Hospital Virgen de la Arrixaca |
| Dokuz Eylul University | Dokuz Eylul University |
| Alder Hey Children’s Hospital | Alder Hey Children’s Hospital^2^ |
| Birmingham Children’s Hospital | Birmingham Children’s Hospital^2^ |
| Bristol Royal Hospital for Children | Bristol Royal Hospital for Children^2^ |
| Evelina Children’s Hospital | Evelina Children’s Hospital^2^ |
| Great North Children’s Hospital–Newcastle | Great North Children’s Hospital–Newcastle^2^ |
| Great Ormond Street | Great Ormond Street^2^ |
| King’s College Hospital | King’s College Hospital^2^ |
| Royal Hospital for Sick Children | Royal Hospital for Sick Children^2^ |
| St George’s Hospital | St George’s Hospital^2^ |
| University Hospital of North Staffordshire NHS Trust | University Hospital of North Staffordshire NHS Trust^2^ |
| University Hospital Southampton NHS Foundation Trust | University Hospital Southampton NHS Foundation Trust^2^ |
| Royal Manchester Children’s Hospital | Royal Manchester Children’s Hospital^2^ |
|  |  |
| **Asia** |  |
| Beijing Children’s Hospital | Beijing Children’s Hospital |
| Advanced Pediatrics | Advanced Pediatrics |
| All India Institute | All India Institute |
| Kyoto Prefectural | Kyoto Prefectural |
| Tokyo Metropolitan | Tokyo Metropolitan |
| Shizuoka Children’s Hospital | Shizuoka Children’s Hospital |
| University Malaya Medical Center | University Malaya Medical Center |
| Kebangsaan Malaysia Medical Center | Kebangsaan Malaysia Medical Center |
| KK Women’s and Children’s Hospital | KK Women’s and Children’s Hospital |
| National University Hospital | National University Hospital |
|  |  |
| **Africa** |  |
| Chris Hani Baragwanath | Chris Hani Baragwanath |
| Rahima Moosa Mother and Child Hospital | Rahima Moosa Mother and Child Hospital |
| Steve Biko Academic Hospital | Steve Biko Academic Hospital |
|  |  |
| **Australia/New Zealand** |  |
| Mater Children’s Hospital | Mater Children’s Hospital |
| Princess Margaret Hospital | Princess Margaret Hospital |
| Royal Children’s Melbourne | Royal Children’s Melbourne |
| Sydney Children’s Hospital | Sydney Children’s Hospital |
| Royal Children’s Brisbane | Royal Children’s Brisbane |
| Children’s Hospital Westmead | Children’s Hospital Westmead |
| Starship Children’s Hospital | Starship Children’s Hospital |

^1^The Children’s Hospital of Philadelphia Institutional Review Board (IRB) served as the central IRB for this study.

^2^The study protocol was reviewed and approved by East of Scotland Research Ethics Service as part of the National Health Service. In addition, all sites in the United Kingdom also obtained local study approval.
